# “I Wish No Child Died Like This”: Analyzing Responses from Parents of Babies Who Died of Complex Congenital Heart Disease in a Pediatric Intensive Care Unit

**DOI:** 10.3390/children12020209

**Published:** 2025-02-10

**Authors:** Francesca Benedetti, Viviana Verzeletti, Simonetta Papa, Luca Giacomelli, Caterina Agosto

**Affiliations:** 1 Department of Women’s and Children’s Health, University of Padova, 35129 Padova, Italy; francesca.benedetti@aopd.veneto.it (F.B.); caterina.agosto@aopd.veneto.it (C.A.); 2 Polistudium SRL, 20121 Milan, Italy; viviana.verzeletti@gmail.com (V.V.); simonetta.papa@polistudium.it (S.P.)

**Keywords:** end-of-life care, pediatric palliative care, congenital heart disease, bereavement, quality of dying and death, qualitative study

## Abstract

Background: The death of a child due to complex congenital heart disease (CCHD) in pediatric intensive care units profoundly affects families, often resulting in lasting grief and emotional distress. Despite advancements in pediatric palliative care (PPC), significant gaps persist in communication and end-of-life (EoL) planning. This study explores the experiences and perceptions of bereaved parents to identify areas for improvement in PPC delivery. Methods: A qualitative phenomenological design was used to analyze the lived experiences of 18 bereaved parents whose children died from CCHD at a tertiary cardiac center. Semi-structured telephone interviews were conducted, incorporating five open-ended questions. Data were analyzed inductively using Colaizzi’s method to identify recurring themes and subthemes. Results: Four key thematic areas emerged: communication issues, the parental role, child care, and bereavement support. Parents highlighted inconsistent communication, lack of preparedness for EoL decisions, and emotional isolation as major challenges. Positive experiences often involved compassionate healthcare providers and structured psychological support. A significant proportion of parents identified family support and faith as key coping mechanisms, while others expressed dissatisfaction with post-mortem follow-up and the absence of long-term bereavement care. Conclusions: Bereaved parents’ experiences underscore the need for improved communication strategies, greater parental involvement in care, and enhanced bereavement support. Integrating structured decision-making pathways early in the care trajectory may help mitigate parental distress and improve the quality of EoL experiences for children with CCHD.

## 1. Introduction

The death of a child is a profound loss that has deep emotional and psychological consequences for families and also poses significant emotional challenges for healthcare professionals (HCPs) [1,2,3]. Pediatric intensive care units (PICUs) often witness such tragedies, particularly in cases involving complex congenital heart disease (CCHD). These settings require a comprehensive integration of pediatric palliative care (PPC) not only to support the child and their family during end-of-life (EoL) care but also to address the emotional challenges faced by HCPs [4,5].

PPC services are pivotal in enhancing EoL care, alleviating distress, and improving outcomes for children, families, and HCPs [1]. Effective PPC relies on honest, compassionate, and ongoing communication tailored to families’ cultural, emotional, and spiritual needs. Rather than a single event, this communication unfolds over time through multiple stages [1,6]. Conducted in private, supportive settings, these discussions help families gradually comprehend their child’s prognosis, establish care goals, and navigate emotional and practical caregiving challenges. The iterative process fosters trust and supports families’ adaptation as the child’s condition evolves. Advance care planning (ACP) complements this process by enabling families to articulate preferences for treatment, symptom management, and the location of EoL care, prioritizing quality of life [7].

CCHDs are a leading cause of infant mortality in intensive care settings [8]. Most deaths occur during infancy, often following prolonged hospital stays and invasive procedures, which intensify parental grief. Previous findings from our group revealed that among children with severe CCHD who died in our institution, 97% passed away in the PICU setting, frequently intubated and sedated, with only 23% having a parent present at the bedside during EoL [9]. In our experience, this low presence is influenced more by logistical constraints, such as limited space and restricted visitation policies in Italian PICUs, rather than cultural factors, although recent renovations are progressively incorporating dedicated spaces to facilitate greater parental involvement. Moreover, over 60% of parents identified minimizing their child’s suffering as the primary therapeutic goal, yet many recognized the absence of survival chances only shortly before their child’s death. These data highlight significant gaps in communication and decision-making at EoL, which contribute to parental distress. Understanding the experiences and perceptions of parents regarding their child’s EoL care is therefore essential for improving clinical practices and support systems [9,10,11].

Qualitative research is essential for exploring the emotional, social, and spiritual aspects of care as it provides valuable insights into communication issues, family dynamics, and bereavement experiences. Despite recent growth, qualitative research in this field remains insufficient to fully capture the unique challenges families face, particularly regarding complex decision-making [12,13,14].

To address this gap, we analyzed the experiences of bereaved parents whose children died of CCHD at a tertiary cardiac care center (Paediatric Cardiology and Cardiac Surgery Units of the University of Padova). By highlighting both the child’s and the family’s needs during their most vulnerable moments, this qualitative analysis contributes to the evolving development of family-centered practices for CCHD at the EoL in the PICU.

## 2. Methods

### 2.1. Participants and Setting

Bereaved parents of children (<18 years) who died from CCHD following major cardiac surgery at our tertiary cardiac care center (Paediatric Cardiology and Cardiac Surgery Units of the University of Padova, Italy) between 1 January 2009 and 31 December 2012 were invited to participate. Exclusion criteria included non-fully proficient Italian speakers or ongoing litigation with the hospital. Parents were contacted by the child’s cardiovascular surgeon, who explained the study and sought consent. Only the parent identified as the primary caregiver was interviewed. To minimize the risk of exacerbating acute grief reactions, interviews were conducted at least two years after the child’s death, following the recommendation of psychologists specializing in pediatric palliative care. Data collection took place from 2016, allowing sufficient time for parents to process their loss before participation.

The study was conducted within the framework of a pediatric tertiary hospital where a PPC service has been operational since 2003. This service provides comprehensive PPC across inpatient, outpatient, and community settings, with 24/7 access to consultations by physicians and advanced practice nurses.

### 2.2. Study Context

The study design received approval from the Hospital Ethics Committee, with a waiver granted for obtaining parental consent for the review of clinical records. Participation in the study was entirely voluntary for both parents and healthcare professionals. Demographic and clinical information, including the interval between the child’s death and the interview, was extracted from medical records.

This study is part of a larger project aimed at improving the quality of end-of-life (EoL) care for children with CCHD. The overarching research involved a cross-sectional, questionnaire-based study conducted through semi-structured, telephone-administered interviews [9]. This paper specifically focused on data derived from five open-ended questions in the original questionnaire and spontaneous comments provided by parents during interviews (see Table 1). The results of the retrospective medical record review were reported in the previously published article [9].

### 2.3. Qualitative Approach

This study used phenomenological analysis, a qualitative approach ideal for exploring participants’ lived experiences. The methodology focused on understanding complex phenomena from subjective perspectives without relying on pre-existing theories. This approach was employed to examine the experiences of parents who endured the traumatic loss of a child due to CCHD.

To ensure sensitivity and minimize bias, interviewers underwent specialized training by a psychologist, including role-playing exercises and techniques to bracket personal assumptions. Five semi-structured interview questions were collaboratively designed, with input from external contributors to ensure neutrality (Table 1).

Data were collected through telephone-administered interviews, capturing responses to open-ended questions and spontaneous reflections. The interviews were conducted by a medical doctor trained in pediatric palliative care communication, in collaboration with a senior attending physician specializing in pediatric palliative care. This training focused on sensitive communication strategies for bereaved families. The average duration of the interviews was 43 min, ranging from 30 to 90 min. Interviews were scheduled at times convenient for the parents, with prior coordination to ensure that participants felt emotionally prepared and comfortable to engage in the conversation. While no formal protocol was established to manage emotional distress during the interviews, the interviewers received specialized training from a psychologist specializing in pediatric palliative care. In cases where parents became visibly distressed or started crying, interviewers were instructed to maintain a respectful silence, allowing parents time to compose themselves. After such moments, the interviewer would gently inquire whether the parent wished to continue, pause, or stop the interview, ensuring that the process remained sensitive to the participants’ emotional well-being.

Transcripts were reviewed verbatim, preserving participants’ linguistic authenticity. Data were analyzed inductively, identifying recurring themes and clusters. Two researchers independently coded the data, resolving discrepancies through face-to-face discussions with oversight from a third investigator.

### 2.4. Analytical Process

The analysis adhered to Colaizzi’s iterative method [15], ensuring thematic coherence and data saturation. Spontaneous comments were analyzed within a phenomenological framework, prioritizing participants’ linguistic specificity to ensure fidelity to their perspectives. Thematic content analysis, as outlined by Braun and Clarke [16], was supported by manual coding to organize data and ensure systematic derivation of themes and subthemes.

Rigor was maintained through peer debriefing, triangulation, and referential adequacy. While ethical considerations precluded contacting parents for further validation, comprehensive reviews confirmed the findings’ robustness. The final dataset was distilled into conceptual domains and themes, encapsulating the essence of the parental experience. Spontaneous comments collected during the study were analyzed and categorized into recurring themes. Each comment was further classified as positive or negative. Key categories included communication issues, parental role, child care, and bereavement support. The findings were visually summarized in a graph. A radar (spider) chart was chosen to visually represent the study’s findings as it effectively displayed the themes and their classification as either positive or negative [17]. The chart comprised three data series: (1) positive comments, reflecting satisfaction or constructive experiences; (2) negative comments, highlighting areas of concern or dissatisfaction; and (3) total comments, representing the overall count within each thematic domain. This visual format facilitated a clear comparison across domains, emphasizing the balance—or imbalance—between positive and negative feedback within each theme.

### 2.5. Quantitative Analysis

Demographic and clinical data were encoded and analyzed using IBM SPSS Statistics 21.0. Continuous variables, such as age and duration of hospital stay, were summarized using mean, median, and range. Categorical variables, including demographic characteristics and clinical outcomes, were reported as frequencies in both absolute numbers and percentages.

## 3. Results

### 3.1. Participants and Recruitment

During the study period, 30 children died. Among the families, 28 (93%) met the inclusion criteria. Of these, 10 families (36%) could not be contacted, while 18 families (60%) participated in the study. Interviews were conducted five years after the child’s death (median time), with the time interval ranging from 3.5 to 6.5 years.

### 3.2. Patient Characteristics

Eighteen children were included in the study, of whom 56% (10/18) were male. Table 2 depicts their baseline characteristics. The median age at death was 45 days (range: 15 days to 9 months), with all children being under 1 year of age at the time of death. The median length of the final hospital stay was 27 days (range: 10 to 130 days). Half of the children (9/18, 50%) had single-ventricle physiology anatomy.

Most patients (95%, 17/18) died in the cardiac intensive care unit (CICU), while one child died in the pediatric cardiology ward. In the last 72 h of life, the majority of patients (95%, 17 out of 18) were intubated and under sedation, while 77% (14 out of 18) required mechanical circulatory support, including extracorporeal membrane oxygenation (ECMO). Multi-organ failure (MOF) was the leading cause of death, accounting for 72% of cases (13/18).

Modes of death included failed cardiopulmonary resuscitation (CPR) in 11% of cases (2/18) and withholding of treatment in 88% (16/18). Among the latter, three children had documented do-not-resuscitate (DNR) orders, and for them, the pediatric palliative care consultation was requested.

### 3.3. Parent Characteristics

Women composed the majority of participating parents (78%, 14 out of 18), with a median age of 41 years, ranging from 33 to 52 years. In terms of distance from the hospital, 56% of families (10 out of 18) resided between 30 and 100 km away, 33% (6 out of 18) lived over 100 km away, and 11% (2 out of 18) were located within 30 km of the hospital.

In terms of religious background, 72% of parents (13/18) identified as Catholic, 17% (3/18) identified as Islamic, and 11% (2/18) did not report their religious affiliation. At the time of their child’s death, 11% of parents (2/18) were present.

### 3.4. Open-Ended Questions

When parents were asked, “What was most difficult for the child?” two themes emerged most frequently: the lack of affection due to limited parental contact during hospitalization and the burden of invasive treatments, both identified by 44% of participants (8/18). A smaller proportion of parents (11%, 2/18) mentioned the child’s inability to engage in interactive and playful exchanges typical of early infancy—such as smiling, cooing, and eye contact—as another significant difficulty.

Regarding their own challenges, parents’ responses reflected a range of struggles. Common themes included feeling isolated from extended family, difficulties managing family responsibilities, and the challenges of being far from home, each reported by 17% of participants (3/18). For some parents, the most difficult aspects were their inability to spend time with the child (11%, 2/18), being separated from their other children (11%, 2/18), or lacking time for self-care (11%, 2/18). A few parents also noted the distressing ICU environment (6%, 1/18), feelings of guilt (6%, 1/18), or a profound sense of helplessness in their inability to support their child effectively (6%, 1/18).

When asked, “Is there anything that made things easier?” many parents highlighted the importance of family support (44%, 8/18) and their religious faith (33%, 6/18) as sources of strength. Additionally, a smaller number of participants found solace in their love for the child (11%, 2/18) and maintaining hope (11%, 2/18) during these difficult times.

Parents were also invited to share advice for other families facing similar situations. More than half (56%, 10/18) expressed that there is no universal advice for such circumstances, underscoring the unique and deeply personal nature of each family’s experience. Others suggested seeking clarification from medical staff (11%, 2/18), relying on prayer (17%, 3/18), or consulting a therapist for emotional support (6%, 1/18). A few parents emphasized the importance of avoiding false optimism (6%, 1/18) and focusing on what is best for the child, even if that means accepting the inevitability of death (6%, 1/18).

Finally, parents offered advice to physicians based on their experiences. Many urged healthcare professionals to improve their human connection with families (44%, 8/18) and to provide clear, honest communication when discussing diagnoses (33%, 6/18). Some parents also emphasized the need for more opportunities to dialogue with medical staff (17%, 3/18) and encouraged physicians to give their utmost effort and dedication in supporting families during such difficult times (6%, 1/18).

### 3.5. Spontaneous Comments

Parents’ spontaneous comments revealed a spectrum of positive and negative experiences across four key domains: communication issues, the role as parents, care of the child, and care of bereavement. Positive reflections often emphasized compassionate interactions with healthcare professionals, moments of emotional connection, and support from family or spiritual advisors. However, many parents also highlighted significant challenges, including fragmented communication, feelings of helplessness, and inadequate preparation for their child’s EoL care. The negative comments were particularly concentrated in areas of communication and care management, underscoring gaps in transparency and emotional support. Table 3 provides an overview of these comments classified as either positive or negative and categorized in themes.

**Figure 1:**
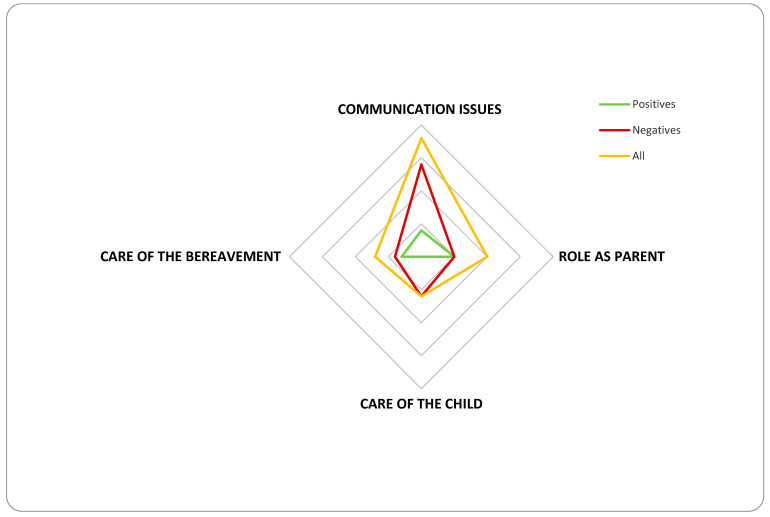
Spider chart of distribution of comments among themes. Positive comments reflect instances of satisfaction or constructive experiences shared by parents. Negative comments highlight areas of concern or dissatisfaction based on parental feedback. All comments represent the overall count of positive and negative comments combined within each thematic domain.

Figure 1 illustrates the distribution of categorized comments, with negative feedback shown in red, positive feedback in green, and the overall totals in yellow. This visual representation highlights the proportions within each theme, enabling a clear comparison of positive and negative perceptions across various aspects of care and support.

## 4. Discussion

This study aimed to deepen our understanding of the feelings and needs of bereaved parents whose children died of CCHD in intensive care. While survival rates for congenital heart diseases have improved in recent years, critical or complex forms, such as single ventricle lesions, remain a leading cause of infant mortality. These conditions are often diagnosed prenatally and necessitate intensive, aggressive interventions within the first months of life [4,9].

Despite advancements in medical care, children with CCHD frequently die in intensive care units, often with limited focus on end-of-life (EoL) care [5]. Research indicates a notable reluctance among cardiologists, cardiac surgeons, and intensive care physicians to recognize or acknowledge when a child is nearing or has reached EoL [7]. This hesitation can lead to the continuation of intensive, invasive treatments that may ultimately prove futile, prolonging the child’s suffering and reducing the quality of their remaining life. Addressing this gap is essential for fostering a more compassionate, patient-centered approach that prioritizes comfort and dignity in critical moments [1,9].

Qualitative studies, such as this, provide a crucial lens into the nuanced emotional and psychological dimensions of pediatric end-of-life care, offering insights that quantitative data alone cannot capture. By exploring the lived experiences of parents, qualitative studies help identify gaps in communication, barriers to care, and opportunities for improving family-centered interventions. This is particularly significant in pediatric palliative care, where subjective experiences and emotional needs play a central role in shaping care delivery. However, the retrospective nature of this study may have influenced the detail or emotional framing of their responses. Additionally, the extended time between the child’s death and the interviews, although intentional to minimize psychological distress, may have introduced recall bias, potentially affecting the accuracy of parents’ recollections. The choice to conduct interviews by telephone, while offering greater flexibility and facilitating participation for parents living far from the study center, limited our ability to observe non-verbal cues, which could have provided additional emotional context. Furthermore, as this research was conducted in a single-center setting and included only Italian-speaking participants, its findings may not fully capture the experiences of diverse populations, cultures, or healthcare systems. Future studies should be designed to explore these varied perspectives further.

The presented data originated from a time period over a decade ago and were part of a broader quantitative research. While articles on this topic have become increasingly prevalent in the past decade, we believe that our analysis holds particular significance as it takes a qualitative approach, delving deeper into the subject matter.

### 4.1. Insights from Open-Ended Questions

The responses to the open-ended questions yielded nuanced insights into the personal and emotional dimensions of the parental experience, beyond the spontaneous reflections. When asked about the most difficult aspects for their child, parents overwhelmingly cited the burden of invasive treatments and the lack of physical affection, underscoring the emotional toll of prolonged hospital stays. This lack of closeness, reported by 44% of participants, highlights the critical need to reassess visitation policies and promote physical interaction between parents and children, even in intensive care environments.

Similarly, the parental responses regarding their own difficulties emphasized isolation, family separation, and the emotional exhaustion inherent in balancing caregiving with external responsibilities. For 17% of parents, the inability to be physically present for their child at all times or to manage other family obligations amplified their distress. Family-centered interventions address not only the child’s needs but also the broader context of parental well-being.

The open-ended responses further provided insight into resilience factors, with family support (44%) and religious faith (33%) emerging as key coping mechanisms. However, the notable absence of universal advice, as expressed by over half of the participants, suggests that while external support is valuable, the grief experience remains profoundly individualized. This draws attention to the necessity for personalized bereavement care pathways that accommodate diverse coping styles and acknowledge the uniqueness of each family’s journey.

Crucially, the reflections offered by parents on advice for clinicians pointed to actionable areas for improvement. Many urged healthcare providers to foster clearer, more compassionate communication, reflecting themes consistent with those found in the spontaneous comments. The emphasis on the human connection between physicians and families underscores the need for communication training that prioritizes empathy, transparency, and emotional support during difficult conversations.

### 4.2. Insights from Spontaneous Comments

Interviews with parents revealed significant emotional and logistical challenges faced by both children and their families during hospitalization. These challenges were categorized into four key thematic areas: communication issues, role as a parent, care of the child, and care of bereavement.

#### 4.2.1. Communication Issues

As shown in Figure 1, communication was the most frequently mentioned theme, with predominantly negative perceptions. Parents commonly reported difficulty understanding medical terminology, leading to misunderstandings and feelings of intimidation. These barriers often discouraged them from seeking clarification, particularly during end-of-life discussions. Consequently, many parents felt excluded from the decision-making process. Some parents advised others in similar situations to proactively seek clarity from medical staff and not hesitate to ask questions.

While some parents acknowledged that information was occasionally delivered with sensitivity, they frequently described it as inconsistent. The absence of a designated reference physician and conflicting information from different healthcare providers compounded their frustration.

These communication challenges left parents unprepared for the progression and intensity of their child’s care. For many, the end-of-life experience felt sudden and unexpected.

#### 4.2.2. Role as a Parent

The theme of the parental role included a relatively higher proportion of positive reflections compared with other themes. However, many parents expressed a profound sense of helplessness regarding their disrupted role. Some marked the need for greater privacy, while others found solace in sharing experiences with fellow parents.

Perspectives on time management varied: some parents wished to remain by their child’s side all day, while others valued breaks to manage emotional strain. Many emphasized the loss of physical contact, such as holding and feeding their baby, as a deeply felt disruption to their experience of new parenthood.

Several parents reflected on feelings of guilt for persisting in treatment efforts, describing their actions in hindsight as “selfish”. The urgent interventions required for CCHDs often led to early mother–child separation, which many mothers found profoundly distressing, encapsulated by the sentiment “from the womb to the intensive care unit”.

#### 4.2.3. Care of the Child

Care of the child received fewer comments, all of which were negative. While parents acknowledged adequate pain management, they consistently perceived their child’s physical suffering and felt that caregivers often prioritized test results and procedures over recognizing the child’s individuality and inherent value. This sense of neglect contributed to a profound emotional burden, with many parents viewing their child’s death as a release from suffering.

One mother, when asked what advice she would offer to other parents in similar situations, stressed the importance of always prioritizing the child’s best interests, even when that may mean accepting the inevitability of the child’s death.

Families with spiritual beliefs noted feeling respected in their faith and supported in their rituals. They identified this as one of the few, but deeply meaningful, sources of relief during this challenging time.

#### 4.2.4. Care of Bereavement

Bereavement care was predominantly associated with negative perceptions. Parents emphasized the importance of maintaining contact with physicians after their child’s death and appreciated the psychological support provided during the grieving process. Mothers, in particular, sought acknowledgment of their motherhood despite their loss. Addressing these bereavement gaps, alongside early activation of palliative care services, is crucial for improving the quality of care and meeting families’ emotional needs.

#### 4.2.5. Miscellaneous

Some comments did not align with the primary themes but highlighted significant challenges. Parents expressed feelings of isolation due to being far from home and family, along with difficulties balancing responsibilities, such as caring for other children. Many also noted insufficient time for self-care during their child’s hospitalization.

#### 4.2.6. Implications for Clinical Practice

Effective communication must be clear, compassionate, and accessible, fostering an environment where parents feel comfortable seeking clarification without intimidation. These conversations require dedicated time and space and should include the referring clinician for continuity of care and a psychologist for emotional support. When multiple clinicians are involved, prior collaboration is essential to ensure consistent messaging and avoid conflicting information. PICU policies should incorporate training for medical staff on communication strategies specific to critical care. In addition to targeted communication training for current healthcare providers, it is equally important to integrate these competencies into the foundational education of future clinicians. Recent reforms in Italian medical schools have introduced mandatory communication courses designed to enhance the human connection in clinical practice. These courses focus on developing skills for delivering difficult news, such as communicating a poor prognosis, fostering empathy, and supporting families during end-of-life situations, thereby addressing key gaps highlighted in our study. Additionally, creating environments where parents can engage more closely with their child, even during intensive care, can alleviate some of the emotional burdens highlighted in the open-ended responses. Flexible visitation policies and parental involvement in daily care routines may foster a greater sense of agency and connection during the hospitalization period.

In this study, only three patients were referred for PPC consultation with ACP in place. Early activation of PPC is crucial for supporting families and care teams in EoL planning [18].

Parental preferences regarding PICU visitation varied, with some valuing privacy and others finding comfort in shared experiences with fellow parents. A flexible policy that accommodates both individualized visiting schedules and shared spaces for connection may address these differing needs. Support should also include addressing challenges related to prolonged hospital stays by facilitating access to accommodations and assisting parents in managing responsibilities, such as caring for other family members.

Many parents pointed out the distress caused by the lack of physical contact with their child. Facilitating physical closeness, particularly at the EoL, should be a priority. Early PPC involvement can help with advance planning to ensure opportunities for physical connection between parents and their child. The involvement of the PPC team plays a crucial role in post-mortem care. Organizing formal meetings between parents and the care team, a routine practice in pediatric palliative care, offers families vital support in the aftermath of their child’s death. As highlighted in our study, parents explicitly requested such meetings, emphasizing their importance in providing closure and emotional support.

## 5. Conclusions

This study provides valuable insights into the emotional and logistical challenges faced by bereaved parents whose children died of CCHD in a PICU setting. Key findings highlight persistent gaps in communication, parental involvement, and bereavement support, alongside the critical roles of family and faith as coping mechanisms. These findings underscore the importance of integrating early, structured pediatric palliative care interventions to address the unique needs of families during end-of-life care.

Future research should focus on longitudinal studies to better understand the evolving nature of parental grief over time and to identify factors that may influence long-term psychological outcomes. Additionally, evaluating the impact of communication training programs in medical education and residency on end-of-life care practices would provide valuable insights into how such interventions can improve clinician–family interactions. Further studies should also explore the experiences of diverse populations across different cultural and healthcare settings to enhance the generalizability of findings and identify context-specific needs.

Improved communication strategies, flexible visitation policies, and enhanced post-mortem follow-up are essential for fostering compassionate, family-centered care. Addressing these areas can improve the quality-of-care delivery and alleviate the emotional burden on families, offering a roadmap for better support during the most vulnerable moments of their journey.

## Figures and Tables

**Table 1:** Open-ended questions asked during interviews.

Question Number	Question
1	What was most difficult for your child?
2	What was most difficult for you as a parent?
3	Is there anything that made things easier for you during this experience?
4	What advice would you give to other parents in a similar situation?
5	What advice would you give to physicians caring for families like yours?

**Table 2:** Summary of children’s demographic and clinical data.

	Number of Children (N = 18)
Gender	56% male (10/18)
Median age at death	45 days (range: 15 days to 9 months)
Age at death	100% < 1 year
Median length of final hospital stay	27 days (range: 10 to 130 days)
Single-ventricle physiology	50% (9/18)
Place of death	94% (17/18) in CICU; 6% (1/18) in pediatric cardiology ward
Intubated and sedated during EoL	95% (17/18)
Receiving ECMO during EoL	77% (14/18)
Cause of death	72% (13/18) MOF; 11% (2/18) cardiogenic shock; 11% (2/18) respiratory failure; 6% (1/18) cerebrovascular accident
Modes of death	11% (2/18) after failed CPR; 89% (16/18) after withholding treatment
DNR orders	Documented in 3 cases during withholding treatment

**Table 3:** Parents’ spontaneous comments categorized by themes.

Themes	Positive Comments	Negative Comments
Communication issues	- The medical language was fine; there was no problem with comprehension. - The cardiac surgeon was delicate; I understood from his eyes the situation was severe. - A doctor brought us to his office, talked with us, gave us time to process, and stayed silent when necessary. - The beauty of working with the therapist was the freedom to express ourselves honestly.	- Communication was fragmented and inconsistent; we lacked a reference physician. - I wanted to ask for more information, but I didn’t have the courage to do so. - We were told everything was fine, but then our child died. - I wanted transparency, but no one explained the gravity of the situation. - Medical terminology was difficult to understand. - I realized the severity only much later, with hindsight. - The communication didn’t prepare me for how quickly my child would deteriorate. - After the procedure, we didn’t know who to talk to or where to turn. - Conflicting information from different physicians added to the confusion. - No one explained to us that intubation would happen; when my wife saw it, she felt unwell. - No one explained how much suffering was involved; the lack of preparation was overwhelming. - I thought at worst he will be disabled, I accepted this, I would never have thought of such a situation. - I understood that they understood it but didn’t tell us. - I didn’t imagine a normal life, but at least a life.
Role as parents	- When she passed away, we were suffering but at peace; that is important. - Visiting hours were suitable, having the whole day would have been overwhelming. - Sharing with other parents created a strong sense of community. - Permitting our pastor to visit was a great kindness. He embraced me, and it gave me strength. - Being allowed more time with my child made me feel like a parent, even though I couldn’t do much.	- I never held my first child, and I didn’t feel like a mother. - I felt empty and guilty for not understanding the severity of the situation. - We insisted on treatments for ourselves, not for him; we were selfish. - I didn’t realize that the second surgery wouldn’t save him. - Moving from the womb straight to intensive care was devastating.
Care of the child		- The child wasn’t living; being connected to tubes isn’t a life. - They focused only on test results, not on the child’s visible suffering. - During those last days, I saw things no parent should see. - Our children aren’t objects; they deserve respect. - I always had the feeling that the child was in pain. - They were just looking at the tests, I’m not a doctor but I think you have to look at the children in the face.
Care of bereavement	- A doctor came to the mortuary; it was a beautiful gesture I will never forget. - I received a condolence letter from a physician, which meant a lot to me. - Joining a support group helped me immensely.	- The biggest gap was the lack of post-mortem follow-up. - I felt the absence of the cardiologists who were involved in his care. - For a mother, the most important thing is that the child is remembered, so she can continue to feel like a mother. - After the child’s death, I left my husband to leave everything behind.

## Data Availability

All data are available from the corresponding author upon reasonable request.
